# The symmetric division Szeged index: A novel tool for predicting physical and chemical properties of complex networks

**DOI:** 10.1016/j.heliyon.2025.e42280

**Published:** 2025-01-27

**Authors:** Modjtaba Ghorbani, Zahra Vaziri, Razie Alidehi-Ravandi, Yilun Shang

**Affiliations:** a Department of Mathematics, Faculty of Science, Shahid Rajaee Teacher Training University, Tehran, 16785-163, Iran; b Department of Computer and Information Sciences, Northumbria University, Newcastle upon Tyne, NE1 8ST, UK

**Keywords:** 05C70, 05C07, 05C35, 92E10, Symmetric division Szeged index (SDZ), Vertex degrees, Graph operations, Chemical graph theory

## Abstract

This paper introduces a novel graph invariant, the symmetric division Szeged index (*SDZ*), which generalizes earlier concepts by focusing on vertices positioned closer to an edge's endpoints rather than vertex degrees. It explores several properties and inequalities associated with the *SDZ*-index, offering examples and results for various graph classes, including bipartite graphs, trees, complete graphs, unicycles, distance-balanced graphs, and triangle-free graphs. Additionally, the *SDZ*-index is contrasted with other well-known graph indices. The behavior of the *SDZ*-index under graph operations, such as corona, sum, lexicographic, and Cartesian products, is also examined. The study highlights the index's potential in topological analysis, particularly in revealing statistically significant correlations with the molecular properties of octane isomers. As research progresses, we anticipate further developments and applications that will enhance our comprehension of complex systems and their properties across fields like network science, computer science, and physics.

## 1 Introduction

In recent years, the study of complex systems has garnered considerable attention across disciplines such as physics, chemistry, and biology. These systems possess intricate structures and behaviors that are difficult to capture using traditional analytical methods. As a result, graph theory has gained prominence as a powerful framework for modeling and analyzing these systems. Through graph theory, complex systems can be visualized as networks, with nodes representing entities and edges symbolizing interactions or relationships between them [1].

As a foundational area of mathematics, graph theory plays a critical role in modeling real-world networks and uncovering their complexities. In this work, we introduce an advanced variant of the symmetric division degree index (referred to as the *SDD*-index), known as the symmetric division Szeged index (*SDZ*). This index uses the number of vertices closer to the endpoints of an edge e=uv instead of relying on the vertex degree at *e*'s endpoints to evaluate graph properties.

This paper provides a comprehensive analysis of the *SDZ*-index, examining its mathematical characteristics, computational complexity, and performance across various graph datasets. The main objective in Section 2 and Section 3 is to compute the *SDZ*-index for several well-known graphs and explore its relationship with other graph invariants. We also compare the *SDZ*-index with other widely used graph indices. Additionally, in Section 4, we identify extremal graphs with respect to the *SDZ*-index and establish upper and lower bounds for it. A significant contribution of this work is in Section 5, the derivation of formulas for computing the *SDZ*-index for different graph operations, such as the sum, corona, lexicographic, and Cartesian products of two graphs, which are derived through a case-by-case analysis of edge behaviors in these operations.

Additionally, we explore potential future research directions concerning the *SDZ*-index and its applications in analyzing real-world networks. In the final section, we demonstrate how the *SDZ*-index serves as an effective tool for studying the physical and chemical properties of molecules based on their topological structures, see [2], [3], [4], [5], [6], [7], [8] as well as [9], [10], [11].

The findings from this research offer fresh insights into the behavior of the *SDZ*-index in graphs and its connection to other graph invariants. This opens up new avenues for future research, including examining the *SDZ*-index in other types of graphs or conducting a deeper investigation into its properties. The study of the *SDZ*-index has enhanced our understanding of graph structures, and ongoing research in this area may reveal further insights into its behavior and broader applications in fields such as mathematics and computer science.

## 2 Preliminaries and definitions

The neighborhood $N_{G}(u)$ of a vertex *u* in a graph *G* is defined as the set of vertices that are adjacent to *u* and $d_{G}(u)$ represents the degree of a vertex *u* in *G*, which is the number of edges that are incident to *u*. In other words, $d_{G}(u)=|N_{G}(u)|$. Also, we let $N_{G}(uv)=N_{G}(u)\cap N_{G}(v)$. All considered graphs in this paper are simple and connected.

Here, $N_{v}(e|G)$ represents the set of vertices in the graph *G* that are closer to a vertex *v* than to another vertex *u* connected by an edge *e*. Similarly, we can define $N_{0}(e|G)$ as the set of vertices in *G* that are equidistant from *u* and *v*. The notation $n_{u}(e|G)$ and $n_{0}(e|G)$ represent the cardinality (i.e. the number of elements) of these sets, respectively. If *G* is clear from the context, we may omit the index *G* in the above notations.

The Szeged index [2] of *G* is defined by

$$Sz(G)=\sum_{uv\in E(G)}n_{u}(e)n_{v}(e).$$

The second arithmetic-geometric $\left(AG_{2}\right)$ index of the graph *G* is defined by

$$AG_{2}(G)=\sum_{uv\in E}\frac{n_{u}+n_{v}}{2\sqrt{n_{u}n_{v}}}.$$

The notation d(G) represents the diameter of a graph *G*. The distance between two vertices *u* and *v*, denoted as d(u,v), is the shortest path length between *u* and *v*. We define the reverse Szeged index and the reverse second Zagreb index by the following formulas:

$$RSz(G)=\sum_{uv\in E}\frac{1}{n_{u}n_{v}},$$

and

$$RM_{2}(G)=\sum_{uv\in E}\frac{1}{d(u)d(v)}.$$

In a recent study, Dehmer et al. [12] introduced a new set of graph indices: $M_{1}$, $M_{2}$, and $M_{3}$. These indices make use of partial Hosoya polynomials, which depict the relationship between the distance of a node and all other nodes within a graph. The $M_{1}$ index is the sum of the absolute values of the zeros of the partial Hosoya polynomials, while the $M_{2}$ index is the sum of the square roots of the absolute values of the zeros. The index $M_{3}$ is a measure similar to entropy that takes into account the absolute values of all partial Hosoya polynomials. These indices provide insights into the connectivity of a graph by analyzing the zeros and their absolute values in the partial Hosoya polynomials.

The symmetric division degree index is defined as follows [3]

$$SDD(G)=\sum_{uv\in E}\frac{d^{2}(u)+d^{2}(v)}{d(u)d(v)}.$$

The symmetric division eccentric index is a newly defined graph invariant by the following formula [4], [13]

$$SDE(G)=\sum_{uv\in E}\frac{\varepsilon^{2}(u)+\varepsilon^{2}(v)}{\varepsilon (u)\varepsilon (v)},$$

and ε(u) denotes the eccentricity of vertex *u*.

In [5], the general form of the *SDD* called the generalized symmetric division degree is defined by replacing the degree of vertices with a function of vertex properties, denoted by GSDD. The GSDD of graph *G* with respect to function *f* is as follows

$$GSDD(G)=\sum_{uv\in E}\frac{f(u)}{f(v)}+\frac{f(v)}{f(u)},$$

where f(u) and f(v) denote the values of function *f* at vertices *u* and *v*, respectively.

Following, the *SDD*-index and putting $f(u)=n_{u}$ and $f(v)=n_{v}$, for every edge e=uv, in generalized form of *SDD*-index, the symmetric division Szeged index is defined as follows:

$$SDZ(G)=\sum_{uv\in E}\left(\frac{n_{u}}{n_{v}}+\frac{n_{v}}{n_{u}}\right).$$

A triangulated graph is a planner graph for which all faces are triangles. A triangle-free graph is a simple graph in which no three vertices form a triangle. Also, we say that a graph *G* is distance-balanced (*DB*) when each edge e=uv satisfies $n_{u}(e)=n_{v}(e)$.

Numerous examples and results related to *SDZ*-index have been explored for various graph classes, including bipartite graphs such as trees, complete graphs, unicycle graphs, distance-balanced graphs, and triangle-free graphs. The values of *SDZ* have been determined for these graph types, providing insights into their structural properties. In continuing, we present some examples:

Example 2.1 For every edge *uv* of the star graph $S_{n}$ with d(u)=n−1, it is not difficult to see that $n_{u}=n-1$ and $n_{v}=1$. It follows

$$\frac{n_{u}}{n_{v}}+\frac{n_{v}}{n_{u}}=\frac{n-1}{1}+\frac{1}{n-1}=\frac{n^{2}-2n+2}{n-1}.$$

Summing over all edges $uv\in E(S_{n})$, we get

$$SDZ(S_{n})=\sum_{uv\in E}\left(\frac{n_{u}}{n_{v}}+\frac{n_{v}}{n_{u}}\right)=(n-1)\left(\frac{n^{2}-2n+2}{n-1}\right)=n^{2}-2n+2.$$

Example 2.2 Let $S_{m,n}$ be a bistar graph on n+m+2 vertices. Suppose e=uv is a pendant edge of $S_{m,n}$. Then

$$\frac{n_{u}}{n_{v}}+\frac{n_{v}}{n_{u}}=\frac{1}{m+n+1}+\frac{m+n+1}{1}.$$

For the central edge f=xy, we have

$$\frac{n_{x}}{n_{y}}+\frac{n_{y}}{n_{x}}=\frac{m+1}{n+1}+\frac{n+1}{m+1},$$

hence

$$SDZ(S_{m,n})=\sum_{uv\in E}\left(\frac{n_{u}}{n_{v}}+\frac{n_{v}}{n_{u}}\right)=(m+n)\left(\frac{1}{m+n+1}+\frac{m+n+1}{1}\right)+\left(\frac{m+1}{n+1}+\frac{n+1}{m+1}\right).$$

Example 2.3 Consider the complete graph $K_{n}$. Then for each edge e=uv, we obtain $n_{u}=n_{v}=1$ and thus

$$SDZ(K_{n})=2\left(\frac{n(n-1)}{2}\right)=n^{2}-n.$$

## 3 The *SDZ*-index versus other indices

In this section, we compare *SDZ*-index with other graph indices and present several properties and inequalities associated with *SDZ*.

Consider *m* is the number of edges of a graph *G*. Then we have

$$SDZ(G)=\sum_{uv\in E}\frac{n_{u}^{2}+n_{v}^{2}}{n_{u}n_{v}}=\sum_{uv\in E}\frac{{\left(n_{u}+n_{v}\right)}^{2}-2n_{u}n_{v}}{n_{u}n_{v}}=\sum_{uv\in E}\frac{{\left(n_{u}+n_{v}\right)}^{2}}{n_{u}n_{v}}-2m=4\sum_{uv\in E}{\left(\frac{n_{u}+n_{v}}{2\sqrt{n_{u}n_{v}}}\right)}^{2}-2m<4{\left(\sum_{uv\in E}\frac{n_{u}+n_{v}}{2\sqrt{n_{u}n_{v}}}\right)}^{2}-2m=4{\left(AG_{2}\right)}^{2}(G)-2m.$$

Hence

$$AG_{2}(G)>\frac{\sqrt{SDZ(G)+2m}}{2}.$$

Since

$$AG_{2}(G)=\sum_{uv\in E}\frac{n_{u}+n_{v}}{2\sqrt{n_{u}n_{v}}}=\sum_{uv\in E}\frac{1}{2}\left(\sqrt{\frac{n_{u}}{n_{v}}}+\sqrt{\frac{n_{v}}{n_{u}}}\right)<SDZ(G),$$

we have $SDZ(G)>\frac{\sqrt{SDZ(G)+2m}}{2}$. Then ${\left(SDZ\right)}^{2}(G)>\frac{SDZ(G)+2m}{4}$. It is easy to see that

$${\left(SDZ\right)}^{2}(G)-\frac{1}{4}SDZ(G)+\frac{1}{64}>\frac{m}{2}+\frac{1}{64}.$$

Thus ${\left(SDZ(G)-\frac{1}{8}\right)}^{2}>\frac{m}{2}+\frac{1}{64}$. Therefore we proved the following proposition.

Proposition 3.1 $SDZ(G)>\frac{1}{8}(\sqrt{32m+1}+1)$*.*

In the next section, we improve the above lower bound.

Theorem 3.1 *Let G be a connected triangle-free graph with*n≥3*vertices. Then*$RSz(G)\leq RM_{2}(G)$*with equality if and only if G has diameter two.*

Proof Let e=uv be an arbitrary edge of *G*. Since *G* is triangle-free, it follows that $n_{u}(e)n_{v}(e)\geq d(u)d(v)$. Therefore

$$RSz(G)=\sum_{uv\in E}\frac{1}{n_{u}n_{v}}\leq \sum_{uv\in E}\frac{1}{d(u)d(v)}=RM_{2}(G).$$

Suppose $RSz(G)=RM_{2}(G)$. We claim that d(G)=2. Since $G≇K_{n}$, we obtain d(G)≥2. If d(G)>2, then there exists an induced path $P_{4}=u_{0}u_{1}u_{2}u_{3}$ and for edge $e=u_{0}u_{1}$ it follows that $n_{u_{1}}(e)>d(u_{1})$ (since $d(u_{1},u_{3})=2<3=d(u_{0},u_{3})$), a contradiction. Conversely, let d(G)=2. Then for all vertices *w* that are at a distance greater than one from the vertices *u* and *v* it holds d(v,w)=d(u,w)=2. Finally, $n_{u}(e)n_{v}(e)=d(u)d(v)$. □

Corollary 3.1 *For a bipartite graph G with at least three vertices, the following inequality holds*

$$SDZ(G)\leq n^{2}RM_{2}(G)-2m.$$

*The equality holds if and only if* d(G)=2 *.*

Proof Let *G* be a bipartite graph. Then it is clear that

$$SDZ(G)=\sum_{uv\in E}\frac{n_{u}^{2}+n_{v}^{2}}{n_{u}n_{v}}=\sum_{uv\in E}\frac{{\left(n_{u}+n_{v}\right)}^{2}-2n_{u}n_{v}}{n_{u}n_{v}}=\sum_{uv\in E}\left(\frac{n^{2}}{n_{u}n_{v}}-2\right).$$

Hence $SDZ(G)=n^{2}RSz(G)-2m$. By Theorem 3.1, we have

$$SDZ(G)\leq n^{2}RM_{2}(G)-2m,$$

and equality holds if and only if the diameter of *G* is 2. □

## 4 Bounds on the *SDZ*-index of graphs

This section discusses the computation of the *SDZ*-index of graphs. The section goes on to prove a theorem that gives bounds on the *SDZ*-index of a graph with order *n* and *m* edges. It is demonstrated that the lower bound of *SDZ*-index is equal to twice the number of edges, with equality holding if and only if the graph is distance-balanced. Furthermore, we show that $S_{n}$ has the maximum *SDZ*-index. As a consequence, it can be concluded that the maximum value of the *SDZ*-index for a tree is upper-bounded by $n^{2}-2n+2$. Additionally, we prove that among all trees that are non-isomorphic with $S_{n}$, the graph bistar has the maximum *SDZ*-index, and the graph $P_{n}$ has the minimum *SDZ*-index. A lemma is also proved, stating that the *SDZ*-index of a non-tree graph is greater than or equal to twice the number of vertices, the condition for equality occurs if and only if the graph is isomorphic to a cycle graph. Also, we determine the maximum value of the *SDZ*-index for unicycle graphs. Finally, a theorem is proved for connected triangulated graphs, establishing an upper bound on the *SDZ*-index in relation to the number of triangles, the order, and the size of the graph.

**Fact 1.** Suppose there exist positive integers *a*, *b*, *s*, and *t* such that k=a+b=s+t, where partitions are sorted by increasing order, namely a<b and s≤t such that a<s. Thus, we have 1≤a<b=k−a and 1<s≤t=k−s. Since the function f(x)=x(k−x) for x≤k−x is an increasing function (since 2x≤k, we have $f^{\prime }(x)=k-2x\geq 0$), we infer that ab<st. For example, if k=6, then 6=1+5=2+4=3+3, and 1×5<2×4<3×3. Thus, if k=a+b, then the minimum value of *ab* is k−1.

We can now state and prove the following theorem.

Theorem 4.1 *Consider a graph G with n vertices and m edges. Then*

$$2m\leq SDZ(G)\leq \left(\frac{n^{2}-2n+2}{n-1}\right)m.$$

*Moreover, the left equality holds if and only if G is a distance-balanced graph. Also, the right upper bound holds if and only if G is a star graph. Especially, in the class of trees,* $SDZ(T)\leq n^{2}-2n+2$ *.*

Proof It is straightforward to observe that for any real number *x*, $x+\frac{1}{x}\geq 2$. Therefore, for any edge uv∈E(G), we have

$$\frac{n_{u}}{n_{v}}+\frac{n_{v}}{n_{u}}\geq 2.$$

So we have the left inequality. To prove the right inequality, let e=uv∈E(G). Then

$$SDZ(e)=\frac{n_{u}}{n_{v}}+\frac{n_{v}}{n_{u}}=\frac{n_{u}^{2}+n_{v}^{2}}{n_{u}n_{v}}=\frac{{\left(n_{u}+n_{v}\right)}^{2}-2n_{u}n_{v}}{n_{u}n_{v}}=\frac{{\left(n_{u}+n_{v}\right)}^{2}}{n_{u}n_{v}}-2.$$

By Fact 1. if $n_{u}+n_{v}=n$, then the maximum value of $\frac{{\left(n_{u}+n_{v}\right)}^{2}}{n_{u}n_{v}}$ is $\frac{n^{2}}{n-1}$. Assume f=xy∈E(G), where $n_{x}+n_{y}<n$. So $n_{x}+n_{y}=n-k$, where 1≤k≤n−2. Note that the minimum value of $n_{x}n_{y}$ is equal to $n_{x}+n_{y}-1$. Thus we have

$$\frac{n^{2}}{n-1}-\frac{{\left(n_{x}+n_{y}\right)}^{2}}{n_{x}+n_{y}-1}=\frac{n^{2}(n_{x}+n_{y}-1)-{\left(n_{x}+n_{y}\right)}^{2}(n-1)}{\left(n-1)(n_{x}+n_{y}-1\right)}=\frac{n^{2}(n-k-1)-{\left(n-k\right)}^{2}(n-1)}{\left(n-1)(n_{x}+n_{y}-1\right)}=\frac{kn(n-2)-k^{2}(n-1)}{\left(n-1)(n-k-1\right)}>0.$$

Hence, $\frac{n^{2}}{n-1}-2$ is the maximum value of the SDZ(e), for each edge e∈E(G). Therefore

$$SDZ(G)\leq \left(\frac{n^{2}-2n+2}{n-1}\right)m.$$

Now assume that SDZ(G)=2m. Then, for each edge uv∈E(G), we must have $\frac{n_{u}}{n_{v}}+\frac{n_{v}}{n_{u}}=2$. This implies that $n_{u}=n_{v}$, for all uv∈E(G). Hence, if SDZ(G)=2m, then *G* is distance-balanced.

Conversely, suppose that *G* is a distance-balanced graph. Then $n_{u}=n_{v}$, for all uv∈E(G). Hence, $\frac{n_{u}}{n_{v}}+\frac{n_{v}}{n_{u}}=2$. Therefore, if *G* is a distance-balanced graph, then SDZ(G)=2m.

Consider $SDZ(G)=\left(\frac{n^{2}-2n+2}{n-1}\right)m$. This implies that

$$\frac{n_{u}}{n_{v}}+\frac{n_{v}}{n_{u}}=\frac{n^{2}-2n+2}{n-1}=\frac{n^{2}}{n-1}-2,$$

for all uv∈E(G). Thus, we have $n_{u}=n-1$ and $n_{v}=1$, which means that *uv* is a leaf. The only graph with this property is the star graph $S_{n}$. Conversely, let $G≅S_{n}$. According to Example 2.1, we have $SDZ(S_{n})=n^{2}-2n+2$ which proves the right equality in the theorem. □

Lemma 4.1 *Let G be a graph, and* e=uv∈E(G) *be a pendant edge. Then for any non-leaf edge f in G, we have* SDZ(e)>SDZ(f) *.*

Proof Suppose *G* is a graph, and e=uv∈E(G) is a pendant edge. By Fact 1. and the proof of Theorem 4.1, we can infer that SDZ(e)>SDZ(f) for any non-leaf edge *f* in *G*. □

Theorem 4.2 *Let* $T≇S_{n}$ *be a tree on n vertices. Then we have*

$$SDZ(P_{n})\leq SDZ(T)\leq SDZ(S_{1,n-3})<SDZ(S_{n}).$$

Proof Suppose $T≇S_{n}$ is a tree on *n* vertices, so |E(T)|=n−1. By Lemma 4.1 and Fact 1. it is clear that

$$SDZ(P_{n})\leq SDZ(T)\leq SDZ(S_{1,n-3})<SDZ(S_{n}).$$

 □

Lemma 4.2 *If G is a non-tree graph on n vertices, then* SDZ(G)≥2n *and equality occurs if and only if the graph is isomorphic to a cycle graph* $C_{n}$ *.*

Proof Let e=uv∈E(G). Since $\frac{n_{u}}{n_{v}}+\frac{n_{v}}{n_{u}}\geq 2$, the minimum value of *SDZ* occurs in the distance-balanced graph with the least number of edges. As trees with n>2 vertices are not distance-balanced, the cycle graph $C_{n}$ has the smallest value of *SDZ*-index among all graphs which is 2m=2n. □

Let $U_{n,r}=C_{r}(T_{1},T_{2},...,T_{r})$ be a *n*-vertex unicycle graph with a cycle $C_{r}=v_{1}v_{2}...v_{r}v_{1}$, where 3≤r≤n and $T_{1},T_{2},\ldots ,T_{r}$ be vertex-disjoint trees, with each $T_{i}$ sharing exactly one vertex $v_{i}$ with the cycle $C_{r}$ for i=1,2,…,r, where $|V(T_{i})|\geq 1$. Also, $C_{r}(S_{t_{1}},S_{t_{2}},...,S_{t_{r}})$ indicates a unicycle graph in which $S_{t_{i}}$ is a star that with the central vertex connecting to the vertex $v_{i}\in V(C_{r})$.

Theorem 4.3 *Consider a unicycle graph G on n vertices. Then*

$$2n\leq SDZ(G)\leq (n-3)\left(\frac{n^{2}-2n+2}{n-1}\right)+2\left(\frac{n^{2}-2n+4}{n-2}\right)+2.$$

*Moreover, the left equality holds if and only if G is a cycle graph* $C_{n}$ *. Also, the right upper bound holds if and only if* $G≅C_{3}(S_{n-2})$ *.*

Proof Suppose $G≅U_{n,r}$. According to Lemma 4.2, the left equality holds for a cycle graph $C_{n}$. Assume that $G≇C_{n}$ and e∈E(G) be a leaf and f∈E(G) be a non-leaf edge on a tree connected to $C_{r}$. Therefore by Lemma 4.1 we have SDZ(e)>SDZ(f). Then

$$SDZ(C_{r}(S_{t_{1}},S_{t_{2}},...,S_{t_{r}}))\geq SDZ(C_{r}(T_{1},T_{2},...,T_{r})),$$

where $|V(T_{i})|=t_{i}\geq 1$.

Now, we investigate the following two cases:

**Case 1**. Suppose *r* is even and $g=uv\in E(C_{r})$ is an edge of $C_{r}(S_{t_{1}},S_{t_{2}},...,S_{t_{r}})$. Since

$$SDZ(g)=\frac{{\left(n_{u}+n_{v}\right)}^{2}}{n_{u}n_{v}}-2=\frac{n^{2}}{n_{u}n_{v}}-2,$$

then the maximum value of SDZ(g) occurs when $n_{u}n_{v}$ is minimum. So by Fact 1. we infer that $\left|n_{u}-n_{v}\right|$ should be maximum. It is easy to see that the maximum value of $\left|n_{u}-n_{v}\right|$ happens in $C_{r}(S_{n-r+1})$. Hence

$$SDZ(C_{r}(S_{n-r+1}))\geq SDZ(C_{r}(S_{t_{1}},S_{t_{2}},...,S_{t_{r}})).$$

We claim that

$$SDZ(C_{3}(S_{n-2}))>SDZ(C_{r}(S_{n-r+1})).$$

**Figure 1:**
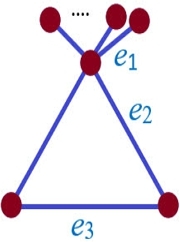
The edges *e*_1_, *e*_2_, and *e*_3_ in *C*_3_(*S*_*n*−2_).

Let $e_{1},e_{2}$, and $e_{3}$ in $E(C_{3}(S_{n-2}))$ as depicted in Fig. 1. Hence $SDZ(e_{1})=\frac{n^{2}}{n-1}-2$, $SDZ(e_{2})=\frac{{\left(n-1\right)}^{2}}{n-2}-2$, and $SDZ(e_{3})=2$. Assume $f_{1}\in E(C_{r}(S_{n-r+1}))$ is a leaf and $f_{2}\in E(C_{r}(S_{n-r+1}))$ is a non-leaf edge. Then we infer that $SDZ(e_{1})=SDZ(f_{1})$ and $SDZ(e_{1})>SDZ(f_{2})$. Therefore we investigate the following inequality

$$SDZ(e_{3})+2SDZ(e_{2})>3SDZ(f_{2}),$$

means that

$$2+2\left(\frac{{\left(n-1\right)}^{2}}{n-2}-2\right)>3\left(\frac{n^{2}}{\left(\frac{n-k}{2}\right)\left(\frac{n-k}{2}+k\right)}-2\right),$$

where *k* is the number of the leaves of $C_{r}(S_{n-r+1})$, and 1≤k≤n−4. Then by Fact 1. we conclude that the maximum value of $SDZ(f_{2})$ occurs if k=n−4. We subtract both sides of the inequality and obtain

$$2+2\left(\frac{{\left(n-1\right)}^{2}}{n-2}-2\right)-3\left(\frac{n^{2}}{\left(\frac{n-k}{2}\right)\left(\frac{n-k}{2}+k\right)}-2\right)\;\;=2+2\left(\frac{n^{2}-4n+5}{n-2}\right)-6\left(\frac{n^{2}+k^{2}}{n^{2}-k^{2}}\right)\;\;=\frac{2n^{2}-6n+6}{n-2}-6\left(\frac{n^{2}+{\left(n-4\right)}^{2}}{n^{2}-{\left(n-4\right)}^{2}}\right)\;\;=\frac{n^{3}-2n^{2}-12n+24}{\left(n-2)(2n-4\right)}>0.$$

Hence we are done.

**Case 2**. Let r>3 be odd and $g=uv\in E(C_{r}(S_{t_{1}},S_{t_{2}},...,S_{t_{r}}))$ is a non-leaf. Since for every $n_{u}+n_{v}$ the maximum value of SDZ(g) occurs when $\left|n_{u}-n_{v}\right|$ is maximum ($n_{u},n_{v}\geq \frac{r-1}{2}$) and the function $f(x)=\frac{x}{y}+\frac{y}{x}$, where x≥y≥1, is increasing, we have

$$SDZ(g)\leq \frac{{\left(n-1\right)}^{2}}{\left(\frac{r-1}{2}\right)\left(\frac{r-1}{2}+k\right)}-2,$$

where k=n−r is the number of leaves, and 1≤k≤n−5. Then we can infer that the maximum value of the *SDZ* contribution occurs for r=5. Similarly to Case 1, let $e_{1},e_{2}$, and $e_{3}$ in $E(C_{3}(S_{n-2}))$ as depicted in Fig. 1 and we show that

$$SDZ(e_{3})+2SDZ(e_{2})>3SDZ(g).$$

Therefore, we subtract both sides of the inequality and obtain

$$2+2\left(\frac{{\left(n-1\right)}^{2}}{n-2}-2\right)-3\left(\frac{{\left(n-1\right)}^{2}}{\left(\frac{5-1}{2}\right)\left(\frac{5-1}{2}+(n-5)\right)}-2\right)\;\;=\frac{2n^{2}-6n+6}{n-2}-\frac{3n^{2}-18n+39}{2n-6}\;\;\;=\frac{n^{3}-27n+42}{\left(n-2)(2n-6\right)}>0.$$

Hence we are done. □

Lemma 4.3 *[14]* *Let G be a simple graph of order n, and suppose that G has* t(G) *triangles. Then*

$$\sum_{uv\in E}|N_{G}(uv)|=3t(G).$$

Theorem 4.4 *Let G be a connected triangulated graph with* n≥2 *vertices, m edges, and* t(G) *triangles. Then*

$$SDZ(G)<n^{2}m+9t^{2}(G)-6nt(G)-2m.$$

Proof Let e=uv∈E(G). We have

$$SDZ(G)=\sum_{e\in E}\frac{{\left(n_{u}+n_{v}\right)}^{2}}{n_{u}n_{v}}-2m.$$

Since $n_{u}+n_{v}\leq n-|N_{G}(uv)|$ and $n_{u}n_{v}\geq 1$, we have

$$SDZ(G)\leq \sum_{e\in E}{\left(n-|N_{G}(uv)|\right)}^{2}-2m\leq n^{2}m+\sum_{e\in E}|N_{G}(uv){|}^{2}-2n\sum_{e\in E}|N_{G}(uv)|-2m<n^{2}m+{\left(\sum_{e\in E}|N_{G}(uv)|\right)}^{2}-2n\sum_{e\in E}|N_{G}(uv)|-2m=n^{2}m+9t^{2}(G)-6nt(G)-2m.$$

 □

## 5 *SDZ*-index of graph products

In this section, we investigate the *SDZ*-index of various graph products, such as the sum, corona, Cartesian, and lexicographic products of two graphs. We establish certain conditions for two graphs, $G_{1}$ and $G_{2}$, under which the lower bound occurs for the product of $G_{1}$ and $G_{2}$.

To construct the graph sum $G_{1}+G_{2}$, two graphs $G_{1}$ and $G_{2}$ with disjoint vertex sets $V(G_{1})$ and $V(G_{2})$ are combined. The resulting graph has a vertex set of $V(G_{1})\cup V(G_{2})$ and an edge set of $E(G_{1})\cup E(G_{2})\cup \{uv\;|\;u\in V(G_{1}),v\in V(G_{2})\}$. This construction involves connecting each vertex of $G_{1}$ to each vertex of $G_{2}$, while preserving all edges in both graphs.

Given two graphs $G_{1}$ and $G_{2}$, the corona product $G_{1}oG_{2}$ is formed by creating $\left|V(G_{1})\right|$ copies of $G_{2}$ and connecting each vertex of the *i*-th copy with vertex $v_{i}\in V(G_{1})$.

The Cartesian product $G_{1}\times G_{2}$ of two graphs $G_{1}$ and $G_{2}$ has the vertex set $V(G_{1}\times G_{2})=V(G_{1})\times V(G_{2})$ and $\left(u_{i},v_{j})(u_{k},v_{l}\right)$ is an edge of $G_{1}\times G_{2}$, if $u_{i}=u_{k}$ and $v_{j}v_{l}\in E(G_{2})$, or $u_{i}u_{k}\in E(G_{1})$ and $v_{j}=v_{l}$.

The lexicographic product of graphs $G_{1}$ and $G_{2}$, $G_{1}[G_{2}]$, has the vertex set $V(G_{1})\times V(G_{2})$ and for $\left(u_{i},v_{j}\right)$ and $\left(u_{k},v_{l}\right)$ to be considered adjacent, either $u_{i}$ is adjacent to $u_{k}$ or $u_{i}$ is equal to $u_{k}$ and $v_{j}$ is adjacent to $v_{l}$.

We prove the following fact to use it in determining the lower bound of the product of two graphs.

**Fact 2.** For integers *a*, *b*, and *c*, if a≥b>c≥0, then we show that

(1) $$\frac{a-c}{b-c}+\frac{b-c}{a-c}\geq \frac{a}{b}+\frac{b}{a}.$$

If a=b or c=0, then equality holds. So we consider a>b>c>0 and subtract both sides of the inequality (1). We have

$$\frac{a-c}{b-c}+\frac{b-c}{a-c}-\frac{a}{b}-\frac{b}{a}=\frac{c(a^{3}+b^{3}+2abc-a^{2}b-ab^{2}-a^{2}c-b^{2}c)}{ab(b-c)(a-c)}.$$

Since c>0 and ab(b−c)(a−c)>0, then we should prove that

$$a^{3}+b^{3}+2abc-a^{2}b-ab^{2}-a^{2}c-b^{2}c>0.$$

We have

$$a^{3}+b^{3}+2abc-a^{2}b-ab^{2}-a^{2}c-b^{2}c\;\;\;=(a^{3}-a^{2}b)+(b^{3}-ab^{2})+(2abc-a^{2}c-b^{2}c)\;\;=a^{2}(a-b)-b^{2}(a-b)-c(a^{2}+b^{2}-2ab)\;\;=(a-b)(a^{2}-b^{2})-c{\left(a-b\right)}^{2}\;\;\;=(a-b)(a^{2}-b^{2}-ca+cb).$$

As a>b, there is integer x>0 such that a=b+x. Hence

$$a^{2}-b^{2}-ca+cb={\left(b+x\right)}^{2}-b^{2}-c(b+x)+cb=x^{2}+2bx-cx>0,$$

and we are done.

Lemma 5.1 *For a graph G, the equation*

$$\sum_{uv\in E}\frac{d_{G}(u)-|N_{G}(uv)|}{d_{G}(v)-|N_{G}(uv)|}+\frac{d_{G}(v)-|N_{G}(uv)|}{d_{G}(u)-|N_{G}(uv)|}=SDD(G)$$

*holds if and only if, for every edge*e=uv∈E(G)*,*$d_{G}(u)=d_{G}(v)$*or when*$d_{G}(u)\neq d_{G}(v)$*, then*$|N_{G}(uv)|=0$*.*

Proof Suppose e=uv∈E(G). If $d_{G}(u)=d_{G}(v)$, then

$$\frac{d_{G}(u)-|N_{G}(uv)|}{d_{G}(v)-|N_{G}(uv)|}+\frac{d_{G}(v)-|N_{G}(uv)|}{d_{G}(u)-|N_{G}(uv)|}=2,$$

and

$$\frac{d_{G}(u)}{d_{G}(v)}+\frac{d_{G}(v)}{d_{G}(u)}=2.$$

Now consider $d_{G}(u)\neq d_{G}(v)$ and $|N_{G}(uv)|=0$. Then since

$$\frac{d_{G}(u)-|N_{G}(uv)|}{d_{G}(v)-|N_{G}(uv)|}+\frac{d_{G}(v)-|N_{G}(uv)|}{d_{G}(u)-|N_{G}(uv)|}=\frac{d_{G}(u)}{d_{G}(v)}+\frac{d_{G}(v)}{d_{G}(u)},$$

we are done. Conversely, assume

$$\sum_{uv\in E}\frac{d_{G}(u)-|N_{G}(uv)|}{d_{G}(v)-|N_{G}(uv)|}+\frac{d_{G}(v)-|N_{G}(uv)|}{d_{G}(u)-|N_{G}(uv)|}=SDD(G).$$

So according to inequality (1) for every edge e=uv∈E(G), we have

$$\frac{d_{G}(u)-|N_{G}(uv)|}{d_{G}(v)-|N_{G}(uv)|}+\frac{d_{G}(v)-|N_{G}(uv)|}{d_{G}(u)-|N_{G}(uv)|}=\frac{d_{G}(u)}{d_{G}(v)}+\frac{d_{G}(v)}{d_{G}(u)}.$$

So

$$\frac{d_{G}(u)-|N_{G}(uv)|}{d_{G}(v)-|N_{G}(uv)|}=\frac{d_{G}(u)}{d_{G}(v)},$$

and thus either $d_{G}(u)=d_{G}(v)$ or if $d_{G}(u)\neq d_{G}(v)$, then $|N_{G}(uv)|=0$ holds. □

Theorem 5.1 *Let* $G_{1}$ *and* $G_{2}$ *be two graphs of orders* $n_{1}$ *and* $n_{2}$ *, respectively. Then*

(2) $$SDZ(G_{1}+G_{2})\geq SDD(G_{1})+SDD(G_{2})+2n_{1}n_{2}.$$

*Moreover, the equality holds if and only if* $G_{1}$ *and* $G_{2}$ *are k-regular and* $k^{\prime }$ *-regular graphs, respectively, and* $n_{1}-k=n_{2}-k^{\prime }$ *.*

Proof To compute $SDZ(G_{1}+G_{2})$, we need to evaluate the sum

$$SDZ(G_{1}+G_{2})=\sum_{uv\in E(G_{1}+G_{2})}\frac{n_{u}}{n_{v}}+\frac{n_{v}}{n_{u}}.$$

By the definition of the sum of two graphs, we know that for an edge e=uv in $E(G_{1}+G_{2})$ one of three following cases holds:

**Case 1.***u* and *v* are both in $G_{1}$. Let $w\in V(G_{2})$. Then we have $d_{G_{1}+G_{2}}(u,w)=d_{G_{1}+G_{2}}(v,w)=1$. On the other hand, if $w\in V(G_{1})$ (where w≠u,v), $d_{G_{1}}(u,w)>1$ and $d_{G_{1}}(v,w)>1$, then $d_{G_{1}+G_{2}}(u,w)=d_{G_{1}+G_{2}}(v,w)=2$. Thus, we can infer that

$$N_{u}(e)=\{u\}\cup \left[N_{G_{1}}(u)-\left(\{v\}\cup N_{G_{1}}(uv)\right)\right],$$

and

$$N_{v}(e)=\{v\}\cup \left[N_{G_{1}}(v)-\left(\{u\}\cup N_{G_{1}}(uv)\right)\right].$$

**Case 2.***u* and *v* are both in $G_{2}$. This case can be computed similarly to Case 1.

**Case 3.***u* is in $G_{1}$ and *v* is in $G_{2}$. If $w\in V(G_{2})\cup N_{G_{1}}(u)$, then $d_{G_{1}+G_{2}}(u,w)=1$, and all other vertices of $V(G_{1}+G_{2})$ are at distance two from *u*. Similarly, if $w\in V(G_{1})\cup N_{G_{2}}(v)$, then $d_{G_{1}+G_{2}}(v,w)=1$, and all other vertices of $V(G_{1}+G_{2})$ are at distance two from *v*. Therefore, we can infer

$$N_{u}(e)=\{u\}\cup \left(V(G_{2})-(\{v\}\cup N_{G_{2}}(v)\right),$$

and

$$N_{v}(e)=\{v\}\cup \left(V(G_{1})-(\{u\}\cup N_{G_{1}}(u)\right).$$

By combining the number of sets in these cases, we obtain

$$SDZ(G_{1}+G_{2})=\sum_{uv\in E(G_{1})}\frac{d_{G_{1}}(u)-|N_{G_{1}}(uv)|}{d_{G_{1}}(v)-|N_{G_{1}}(uv)|}+\frac{d_{G_{1}}(v)-|N_{G_{1}}(uv)|}{d_{G_{1}}(u)-|N_{G_{1}}(uv)|}\;\;\;+\sum_{uv\in E(G_{2})}\frac{d_{G_{2}}(u)-|N_{G_{2}}(uv)|}{d_{G_{2}}(v)-|N_{G_{2}}(uv)|}+\frac{d_{G_{2}}(v)-|N_{G_{2}}(uv)|}{d_{G_{2}}(u)-|N_{G_{2}}(uv)|}\;\;\;+\sum_{u\in V(G_{1}),v\in V(G_{2})}\frac{n_{2}-d_{G_{2}}(v)}{n_{1}-d_{G_{1}}(u)}+\frac{n_{1}-d_{G_{1}}(u)}{n_{2}-d_{G_{2}}(v)}.$$

Hence by inequality (1) and $\frac{n_{2}-d_{G_{2}}(v)}{n_{1}-d_{G_{1}}(u)}+\frac{n_{1}-d_{G_{1}}(u)}{n_{2}-d_{G_{2}}(v)}\geq 2$, we can infer that the desired result.

Now suppose equality holds in inequality (2). Therefore by Lemma 5.1, for every edge $e=uv\in E(G_{i})$, we have $d_{G_{i}}(u)=d_{G_{i}}(v)$ or $|N_{G_{i}}(uv)|=0$, where i=1,2. On the other hand, since

$$\frac{n_{2}-d_{G_{2}}(v)}{n_{1}-d_{G_{1}}(u)}+\frac{n_{1}-d_{G_{1}}(u)}{n_{2}-d_{G_{2}}(v)}=2,$$

for every $u\in V(G_{1})$ and $v\in V(G_{2})$, we have $n_{1}-d_{G_{1}}(u)=n_{2}-d_{G_{2}}(v)$. Therefore, $G_{1}$ and $G_{2}$ are regular graphs. Conversely, if $G_{1}$ and $G_{2}$ are *k*-regular and $k^{\prime }$-regular graphs, respectively, and $n_{1}-k=n_{2}-k^{\prime }$, then proof is clear. □

Theorem 5.2 *If* $G_{1}$ *and* $G_{2}$ *are two graphs with orders* $n_{1}$ *and* $n_{2}$ *, respectively, then*

(3) $$SDZ(G_{1}\circ G_{2})\geq SDZ(G_{1})+n_{1}SDD(G_{2})+n_{1}n_{2}\left(n_{1}+n_{1}n_{2}-n_{2}+\frac{1}{n_{1}+n_{1}n_{2}-n_{2}}\right).$$

*Furthermore, the equality holds true if* $G_{2}$ *is a complete graph.*

Proof To compute $SDZ(G_{1}\circ G_{2})$, we need to evaluate the sum

$$SDZ(G_{1}\circ G_{2})=\sum_{uv\in E(G_{1}\circ G_{2})}\frac{n_{u}}{n_{v}}+\frac{n_{v}}{n_{u}}.$$

For an edge e=uv in $G_{1}\circ G_{2}$ one of three following cases holds:

**Case 1.***u* and *v* are both in $G_{1}$. In this case, we have

$$n_{u}(e|G_{1}\circ G_{2})=n_{u}(e|G_{1}).n_{2},$$

and

$$n_{v}(e|G_{1}\circ G_{2})=n_{v}(e|G_{1}).n_{2}.$$

**Case 2.***u* and *v* are both in $G_{2}$. In this case, all vertices in $G_{1}$ and other copies of $G_{2}$ are in $N_{0}(e)$. It is easy to see that

$$N_{u}(e)=\{u\}\cup \left[N_{G_{2}}(u)-\left(\{v\}\cup N_{G_{2}}(uv)\right)\right],$$

and

$$N_{v}(e)=\{v\}\cup \left[N_{G_{2}}(v)-\left(\{u\}\cup N_{G_{2}}(uv)\right)\right].$$

**Case 3.***u* is in $G_{1}$ and *v* is in $G_{2}$. In this case, since *u* is adjacent to all vertices of a copy of $G_{2}$ containing *v*, we have $n_{v}=1$ and

$$n_{u}=|V(G_{1})|+|V(G_{2})|\times |V(G_{1})-1|+|V(G_{2})-\left(\{v\}\cup N_{G_{2}}(v)\right)|.$$

By combining the number of sets in these cases, we obtain

$$SDZ(G_{1}\circ G_{2})=\sum_{uv\in E(G_{1})}\frac{n_{u}.n_{2}}{n_{v}.n_{2}}+\frac{n_{v}.n_{2}}{n_{u}.n_{2}}\;\;+n_{1}\sum_{uv\in E(G_{2})}\frac{d_{G_{2}}(u)-|N_{G_{2}}(uv)|}{d_{G_{2}}(v)-|N_{G_{2}}(uv)|}+\frac{d_{G_{2}}(v)-|N_{G_{2}}(uv)|}{d_{G_{2}}(u)-|N_{G_{2}}(uv)|}\;\;+\sum_{u\in V(G_{1})}\sum_{v\in V(G_{2})}n_{1}+n_{1}n_{2}-d_{G_{2}}(v)-1+\frac{1}{n_{1}+n_{1}n_{2}-d_{G_{2}}(v)-1}.$$

Since $1\leq d_{G_{2}}(v)\leq n_{2}-1$, we can infer that the minimum value of

$$n_{1}+n_{1}n_{2}-d_{G_{2}}(v)-1+\frac{1}{n_{1}+n_{1}n_{2}-d_{G_{2}}(v)-1}$$

is

$$n_{1}+n_{1}n_{2}-n_{2}+\frac{1}{n_{1}+n_{1}n_{2}-n_{2}}.$$

Hence by inequality (1), we conclude the desired result.

Now, let equality hold in inequality (3). By Lemma 5.1, for every edge $e=uv\in E(G_{2})$, we have $d_{G_{2}}(u)=d_{G_{2}}(v)$ or $|N_{G_{2}}(uv)|=0$. On the other hand, since for every $v\in V(G_{2})$ we consider $d_{G_{2}}(v)=n_{2}-1$. Thus, $G_{2}$ is a complete graph. Conversely, the proof is straightforward if $G_{2}$ is a complete graph. □

Theorem 5.3 *If* $G_{1}$ *and* $G_{2}$ *are two graphs with orders* $n_{1}$ *and* $n_{2}$ *, respectively, then*

$$SDZ(G_{1}\times G_{2})=n_{2}.SDZ(G_{1})+n_{1}.SDZ(G_{2}).$$

Proof According to the fact that in $G_{1}\times G_{2}$, there are $n_{2}$ copies of $G_{1}$ and $n_{1}$ copies of $G_{2}$, there are two kinds of edges in $G_{1}\times G_{2}$. So we have

$$SDZ(G_{1}\times G_{2})=\sum_{u\in V(G_{1})}\sum_{xy\in E(G_{2})}\frac{n_{\left(u,x\right)}}{n_{\left(u,y\right)}}+\frac{n_{\left(u,y\right)}}{n_{\left(u,x\right)}}+\sum_{x\in V(G_{2})}\sum_{uv\in E(G_{1})}\frac{n_{\left(u,x\right)}}{n_{\left(v,x\right)}}+\frac{n_{\left(v,x\right)}}{n_{\left(u,x\right)}}=\sum_{u\in V(G_{1})}\sum_{xy\in E(G_{2})}\frac{n_{x}.n_{1}}{n_{y}.n_{1}}+\frac{n_{y}.n_{1}}{n_{x}.n_{1}}+\sum_{x\in V(G_{2})}\sum_{uv\in E(G_{1})}\frac{n_{u}.n_{2}}{n_{v}.n_{2}}+\frac{n_{v}.n_{2}}{n_{u}.n_{2}}=n_{1}\left(\sum_{xy\in E(G_{2})}\frac{n_{x}}{n_{y}}+\frac{n_{y}}{n_{x}}\right)+n_{2}\left(\sum_{uv\in E(G_{1})}\frac{n_{u}}{n_{v}}+\frac{n_{v}}{n_{u}}\right)=n_{1}.SDZ(G_{2})+n_{2}.SDZ(G_{1}).$$

 □

Theorem 5.4 *Consider two graphs* $G_{1}$ *and* $G_{2}$ *with orders* $n_{1}$ *and* $n_{2}$ *, and sizes* $m_{1}$ *and* $m_{2}$ *, respectively. Thus, for* $G_{1}[G_{2}]$ *, we have*

(4) $$SDZ(G_{1}[G_{2}])\geq n_{1}SDD(G_{2})+2m_{1}n_{2}^{2}.$$

*The equality is defined by a specific combination of properties:* $G_{2}$ *must be regular, and* $G_{1}$ *must be distance-balanced.*

Proof Suppose $e=(u,x)(v,y)\in E(G_{1}[G_{2}])$. By the definition of the lexicographic of two graphs, we investigate two following cases:

**Case 1**. Let u=v and $xy\in E(G_{2})$. Therefore the edge *e* is in a copy of $G_{2}$. It is easy to see that all vertices in other copies of $G_{2}$ are equidistant from (u,x) and (u,y). For the vertex $\left(u,z)\in V(G_{1}[G_{2}]\right)$, if $z\notin (N_{G_{2}}(x)\cup N_{G_{2}}(y))$, then d((u,x),(u,z))=d((u,y),(u,z))=2. Hence

$$n_{\left(u,x\right)}=d_{G_{2}}(x)-|N_{G_{2}}(xy)|,$$

and

$$n_{\left(u,y\right)}=d_{G_{2}}(y)-|N_{G_{2}}(xy)|.$$

**Case 2**. Let $uv\in E(G_{1})$ and x=y. Hence the edge *e* is in a copy of $G_{1}$. For the vertex $\left(w,z)\in V(G_{1}[G_{2}]\right)$ (w∉{u,v}), if $w\in N_{u}(uv|G_{1})$, then for every $z\in V(G_{2})$, $\left(w,z)\in N_{\left(u,x\right)}(e\right)$. Also, if $w\in N_{v}(uv|G_{1})$, then for every $z\in V(G_{2})$, $\left(w,z)\in N_{\left(v,x\right)}(e\right)$. In addition, if $w\in N_{0}(uv|G_{1})$, then for every $z\in V(G_{2})$, $\left(w,z)\in N_{0}(e\right)$. For every $z\in V(G_{2})$, the vertex (u,z) is adjacent to (v,x). If $z\in N_{G_{2}}(x)$, then (u,z) is adjacent to (u,x) and thus $\left(u,z)\in N_{0}(e\right)$. If $z\notin N_{G_{2}}(x)$, then $\left(u,z)\in N_{\left(v,x\right)}(e\right)$. Similarly, for the vertex (v,z), if $z\in N_{G_{2}}(x)$, then $\left(v,z)\in N_{0}(e\right)$ and if $z\notin N_{G_{2}}(x)$, then $\left(v,z)\in N_{\left(u,x\right)}(e\right)$. Therefore

$$n_{\left(u,x\right)}=n_{2}n_{u}-d_{G_{2}}(x),$$

and

$$n_{\left(v,x\right)}=n_{2}n_{v}-d_{G_{2}}(x).$$

Now, let x≠y. Similarly, we have $n_{\left(u,x\right)}=n_{2}n_{u}-d_{G_{2}}(y)$ and $n_{\left(v,y\right)}=n_{2}n_{v}-d_{G_{2}}(x)$.

Hence

$$SDZ(G_{1}[G_{2}])=\sum_{u\in V(G_{1})}\sum_{xy\in E(G_{2})}\frac{n_{\left(u,x\right)}}{n_{\left(u,y\right)}}+\frac{n_{\left(u,y\right)}}{n_{\left(u,x\right)}}+\sum_{x,y\in V(G_{2})}\sum_{uv\in E(G_{1})}\frac{n_{\left(u,x\right)}}{n_{\left(v,y\right)}}+\frac{n_{\left(v,y\right)}}{n_{\left(v,x\right)}}=n_{1}\sum_{xy\in E(G_{2})}\frac{d_{G_{2}}(x)-|N_{G_{2}}(xy)|}{d_{G_{2}}(y)-|N_{G_{2}}(xy)|}+\frac{d_{G_{2}}(y)-|N_{G_{2}}(xy)|}{d_{G_{2}}(x)-|N_{G_{2}}(xy)|}+\sum_{x,y\in V(G_{2})}\sum_{uv\in E(G_{1})}\frac{n_{2}n_{u}-d_{G_{2}}(y)}{n_{2}n_{v}-d_{G_{2}}(x)}+\frac{n_{2}n_{v}-d_{G_{2}}(x)}{n_{2}n_{u}-d_{G_{2}}(y)}.$$

In other words, according to inequality (1) and as

$$\frac{n_{2}n_{u}-d_{G_{2}}(y)}{n_{2}n_{v}-d_{G_{2}}(x)}+\frac{n_{2}n_{v}-d_{G_{2}}(x)}{n_{2}n_{u}-d_{G_{2}}(y)}\geq 2,$$

we may conclude that

$$SDZ(G_{1}[G_{2}])\geq n_{1}SDD(G_{2})+2m_{1}n_{2}^{2}.$$

Moreover, let equality hold in inequality (4). By Lemma 5.1, for every edge $e=uv\in E(G_{2})$, we must have $d_{G_{2}}(u)=d_{G_{2}}(v)$ or $|N_{G_{2}}(uv)|=0$. On the other hand, for an edge $\left(u,x)(v,y)\in E(G_{1}[G_{2}]\right)$, where $uv\in E(G_{1})$ we have $n_{2}n_{u}-d_{G_{2}}(y)=n_{2}n_{v}-d_{G_{2}}(x)$. Then $G_{2}$ is a regular graph and $G_{1}$ is a distance-balanced graph. Conversely, if $G_{2}$ is a regular graph and $G_{1}$ is a distance-balanced graph, then the proof is clear. □

## 6 Application in chemistry

We examine the potential applications of the *SDZ*-index in chemistry by analyzing its relationship with various physical and chemical attributes of octane isomers. To achieve this, we computed the *SDZ*, *SDE*, and *SDD*-indices for each compound (see Table 1) (the code for calculating the *SDZ* is presented at the end of the article). The results are presented in Table 2, which displays the correlation coefficients between these indices and various properties of octane isomers.

**Table 1:** Properties of the octane isomers.

Compound	*SDZ*	*SDE*	*SDD*	AcentFac	*S*	$M_{1}$	$M_{2}$	$M_{3}$
1	27.48	14.21	15.00	0.40	111.00	40.68	17.97	96.30
2	31.29	14.37	17.33	0.38	109.84	32.65	15.96	68.47
3	32.36	14.38	16.67	0.37	111.26	34.24	16.42	73.27
4	32.63	14.42	16.67	0.37	109.32	32.74	16.04	68.20
5	36.44	14.40	18.67	0.35	108.02	28.15	14.92	51.99
6	36.17	14.40	19.00	0.34	106.98	28.61	15.02	53.75
7	35.10	14.37	19.67	0.36	105.72	24.60	13.95	40.66
8	37.24	14.43	18.00	0.34	106.59	30.14	15.52	57.77
9	36.17	14.37	21.75	0.34	103.42	29.74	15.23	58.35
10	37.50	14.43	20.50	0.32	104.74	32.82	16.18	67.13
11	33.70	14.40	16.00	0.36	109.43	27.81	14.84	50.79
12	44.86	15.00	27.50	0.26	93.06	9.59	8.76	2.54
13	40.25	14.83	20.67	0.32	102.39	16.27	11.20	18.20
14	41.05	14.83	22.83	0.30	101.31	19.91	12.19	29.84
15	39.98	14.75	24.08	0.30	104.09	17.81	11.65	22.58
16	41.31	14.92	22.25	0.29	102.06	18.97	12.00	26.36
17	38.57	14.92	19.25	0.31	101.48	16.27	11.30	17.50
18	37.50	14.83	18.00	0.33	106.06	15.45	10.98	15.69

**Table 2:** Correlation analysis of *SDZ*-index with other graph invariants.

Selected *TI*	*SDZ*	*SDD*	*SDE*	AcentFac	*S*	$M_{1}$	$M_{2}$	$M_{3}$
*SDZ*	1	0.88	0.85	-0.97	-0.90	-0.87	-0.87	-0.87
*SDD*	0.88	1	0.68	-0.89	-0.91	-0.72	-0.74	-0.71
*SDE*	0.85	0.68	1	-0.87	-0.80	-0.93	-0.94	-0.91
AcentFac	-0.97	-0.89	-0.87	1	0.92	0.85	0.85	0.84
*S*	-0.90	-0.91	-0.80	0.92	1	0.82	0.83	0.80
$M_{1}$	-0.87	-0.72	-0.93	0.85	0.82	1	0.99	0.99
$M_{2}$	-0.87	-0.74	-0.94	0.85	0.83	0.99	1	0.99
$M_{3}$	-0.87	-0.71	-0.91	0.84	0.80	0.99	0.99	1

*TI*=Topological index.

**Figure 2:**
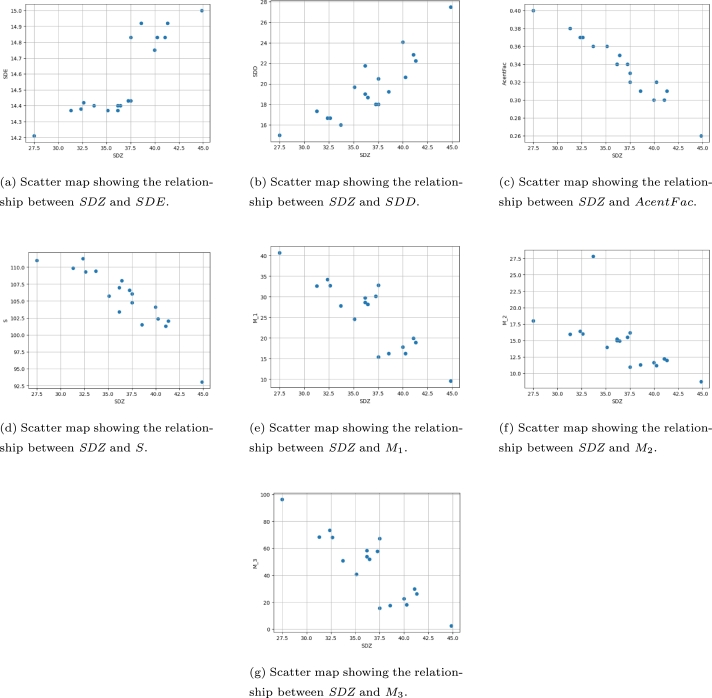
Scatter plots demonstrate the correlation between *SDZ* and different graph properties.

The correlation analysis presented in Table 2 shows the relationship between the *SDZ*-index and other graph invariants. The analysis reveals that the *SDZ*-index has a strong negative correlation with the Acentric Factor and entropy (*S*) indices, as well as with the first, second, and third partial Hosoya polynomials represented by $M_{1}$, $M_{2}$, and $M_{3}$. This indicates that molecules with higher *SDZ*-indices tend to have lower values of these indices. The scatter plot illustrating this correlation is presented in Fig. 2(a-g).

These correlations are significantly higher than those observed for the *SDD*-index, indicating that proximity to edges is a more reliable indicator of these properties than vertex degree alone.

This finding suggests that the *SDZ*-index may provide a complementary perspective to traditional degree-based indices in chemical graph theory. While indices based on degrees are commonly used to predict properties such as reactivity and solubility, they may not capture the full complexity of molecular structure. By considering proximity to edges instead of just vertex degrees, the *SDZ*-index may provide a more nuanced understanding of molecular properties.

The strong correlation between the *SDZ*-index and octane isomer properties suggests that this index can be used to predict these properties for new compounds with high accuracy. This has important implications for chemical engineering and materials science, as it allows for more accurate predictions of compound behavior without the need for expensive and time-consuming experimental testing.

In addition, our analysis also suggests that the *SDZ*-index can provide insights into the structural features that contribute to these properties. By examining the distribution of vertices closer to edges in compounds with different properties, we can identify structural patterns that are associated with specific behaviors. This can inform future research into compound design and optimization for specific applications.

## 7 Conclusion

In conclusion, the introduction of the symmetric division Szeged index offers a novel approach to graph theory, expanding upon the symmetric division degree index by focusing on vertices nearer to the endpoints of an edge rather than just their vertex degrees. We have discussed several properties and inequalities associated with the *SDZ*-index, alongside examples and results for various graph classes, including complete graphs, bipartite graphs, trees, and distance-balanced graphs. Furthermore, we analyzed the behavior of the *SDZ*-index under different graph operations, such as corona, sum, lexicographic, and Cartesian products. The *SDZ*-index also shows a strong connection to other well-known graph invariants, such as the Szeged index and the second Zagreb index.

Our analysis has further revealed that the *SDZ*-index holds promise for applications in chemical graph theory, particularly in predicting the physical and chemical properties of compounds based on their topological structures. This is substantiated by our results, which show a strong correlation between the *SDZ*-index and various physical and chemical properties of octane isomers.

Further research could delve deeper into the properties of the *SDZ*-index, as well as explore its potential applications in other areas, such as network science, computer science, and physics. As our knowledge of this index expands, we anticipate further improvements and applications that will enhance our comprehension of complex systems and their characteristics.

## CRediT authorship contribution statement

**Modjtaba Ghorbani:** Writing – review & editing, Writing – original draft, Software, Conceptualization. **Zahra Vaziri:** Software, Methodology, Investigation. **Razie Alidehi-Ravandi:** Writing – original draft, Software, Resources, Methodology, Investigation. **Yilun Shang:** Writing – review & editing, Supervision, Software, Funding acquisition.

## Declaration of Competing Interest

The authors declare that they have no known competing financial interests or personal relationships that could have appeared to influence the work reported in this paper.

## Figures and Tables

**Figure:**
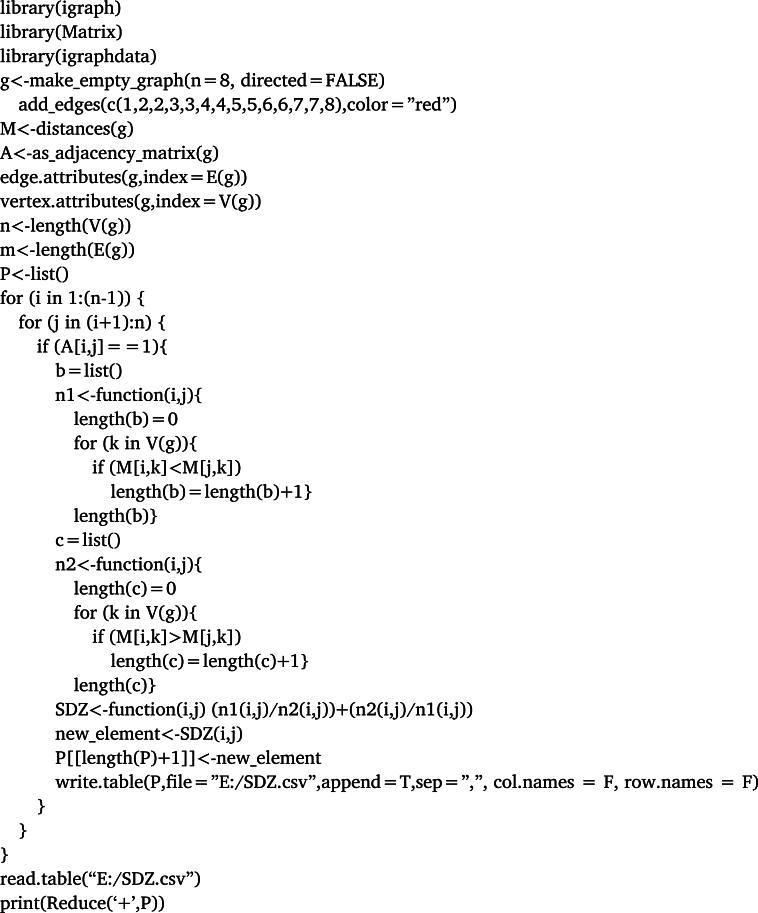


## Data Availability

The data that support the findings of this study are available from the corresponding author upon reasonable request.

## Appendix A

An *R* program for computing the *SDZ*-index

Here, we provide an *R*-code to compute the *SDZ*-index for a given graph.
